# Use of Pressure-Measuring Insoles to Characterize Gait Parameters in Simulated Reduced-Gravity Conditions

**DOI:** 10.3390/s21186244

**Published:** 2021-09-17

**Authors:** Christian Ison, Connor Neilsen, Jessica DeBerardinis, Mohamed B. Trabia, Janet S. Dufek

**Affiliations:** 1 Department of Kinesiology and Nutrition Sciences, University of Nevada, Las Vegas, NV 89154, USA; janet.dufek@unlv.edu; 2 Department of Mechanical Engineering, University of Nevada, Las Vegas, NV 89154, USA; connor.neilsen@colorado.edu (C.N.); deberardinis.jessica@gmail.com (J.D.); mohamed.trabia@unlv.edu (M.B.T.)

**Keywords:** biomechanics, gait analysis, gait characteristics, pressure-measuring insoles, anti-gravity treadmill, reduced-gravity exercise, center of pressure, stance time

## Abstract

Prior researchers have observed the effect of simulated reduced-gravity exercise. However, the extent to which lower-body positive-pressure treadmill (LBPPT) walking alters kinematic gait characteristics is not well understood. The purpose of the study was to investigate the effect of LBPPT walking on selected gait parameters in simulated reduced-gravity conditions. Twenty-nine college-aged volunteers participated in this cross-sectional study. Participants wore pressure-measuring insoles (Medilogic GmBH, Schönefeld, Germany) and completed three 3.5-min walking trials on the LBPPT (AlterG, Inc., Fremont, CA, USA) at 100% (normal gravity) as well as reduced-gravity conditions of 40% and 20% body weight (BW). The resulting insole data were analyzed to calculate center of pressure (COP) variables: COP path length and width and stance time. The results showed that 100% BW condition was significantly different from both the 40% and 20% BW conditions, *p* < 0.05. There were no significant differences observed between the 40% and 20% BW conditions for COP path length and width. Conversely, stance time significantly differed between the 40% and 20% BW conditions. The findings of this study may prove beneficial for clinicians as they develop rehabilitation strategies to effectively unload the individual’s body weight to perform safe exercises.

## 1. Introduction

Simulated reduced-gravity exercise is widely used for rehabilitating patients and athletes with the intention to facilitate early mobility while simultaneously adhering to safe aerobic exercise [1,2] and providing partial body weight support to perform simulated reduced-gravity exercise. The most common methods include the use of water immersion systems, also referred to as water or pool therapy, and harness suspension treadmill systems. Water immersion rehabilitation programs (pool therapy) incorporate movement when the body is partially submerged in water. Thus, a decrease in compressive forces on the musculoskeletal system due to the presence of buoyant forces acting in opposition to the body submerged in water is observed [3,4]. Another approach to unload partial body weight is to use a harness suspension treadmill system to vertically lift the individual partially off the ground [5]. A more recently developed method to provide partial body weight support during exercise is the use of a lower-body positive-pressure treadmill (LBPPT), which contains the lower body in an air-tight, increasingly pressurized air chamber to provide a lift force that effectively reduces musculoskeletal loading, i.e., body weight loading. The use of LBPPT is suggested to benefit those specifically recovering from lower extremity injuries and impairments. LBPPT exercise allows individuals and patients to perform safe aerobic exercises such as walking and running [2,4].

Prior reports have identified kinetic differences when comparing LBPPT walking with over-ground or normal-gravity condition walking. Researchers suggest that the clinical rehabilitative benefits for simulated reduced-gravity exercise include decreased ground reaction forces, increased lower extremity muscular strength, decreased lower extremity pain, and decreased overall exercise recovery time [6,7,8,9]. Although there is growing evidence that exercise performed in these reduced-gravity environments can be an alternative to exercise in normal gravity due to the capability of unloading a portion of body weight, the extent to which LBPPT walking alters kinematic gait characteristics is not well understood. It can be hypothesized that increasing the level of partial body weight support may potentially hinder gait stability due to changes in walking strategy and movement characteristics. Unstable gait leads to a compromised ability to control the position of the body’s center of mass (COM) relative to the base of support (BOS) and is typically observed in individuals with neurological and musculoskeletal disorders [10,11]. Furthermore, recommendations of the optimal magnitude of partial body weight support that ensure non-alteration of gait were not studied [4,6,12,13,14,15,16,17]. In general, the effect of LBPPT on the potential deleterious gait characteristics has yet to be explored. 

Center of pressure (COP) has been utilized as a means for assessing static and dynamic postural stability during gait analysis experiments [18,19]. COP, when examining kinetic plantar pressure measures, is typically used to identify and assess any issues with an individual’s foot pathology during gait. The calculated COP location and its movement during the stance phase of each cycle is reported as an indicator of marked gait stability during dynamic tasks such as walking [20,21]. It was suggested that the assessment of plantar pressure could be an appropriate substitute for measuring foot trauma. In addition, COP path length and width have also been examined to determine preventative fall risk strategies, typically among geriatric and clinical populations [22,23]. Currently, pressure mats and force platforms are typically used to measure COP [19]. Recently, pressure-measuring insoles have been suggested as an acceptable portable alternative to the existing gait analysis instruments [19]. Although pressure-measuring insoles typically sample at a lower frequency compared to force platforms, these frequencies are appropriate for measuring walking kinetics [24]. Pressure-measure insoles can also be used in various environments to record multiple consecutive steps, e.g., anti-gravity treadmill. Therefore, the purpose of our research was to investigate the effect of LBPPT walking on selected gait parameters in simulated reduced-gravity conditions. Specifically, we sought to explore changes in COP path length and width among normal and reduced body weight conditions to assess walking stability. 

We hypothesized that (1) COP path length and width will change as gravity conditions are reduced, and (2) stance time measures will be identical across gravity conditions, as walking speed will be normalized for each participant performing the experiment. The findings from this study may provide additional information for clinicians and researchers in the rehabilitation and exercise prescription of LBPPT walking for those with impaired lower extremity function.

## 2. Materials and Methods

### 2.1. Participants

A convenience sample of 29 healthy, college-aged (24.7 ± 4.0 years, 11 male, 18 female, mass = 72.4 ± 13.5 kg, height = 1.67 ± 0.04 m) (Table 1) volunteers was recruited for this experiment. Participants provided institutionally approved written consent (IRB # 1510671-2) to participate. The inclusion criteria were (a) apparently healthy individuals between 18 and 55 years old, (b) no injuries or medical conditions that would limit the participant from being able to walk unsupported for 12 min, (c) not pregnant or possibly pregnant, and (d) shoe size between 35 and 43 and not greater than 46 (European sizing), due to pressure-measuring insole size availability.

### 2.2. Instrumentation

**Figure 1:**
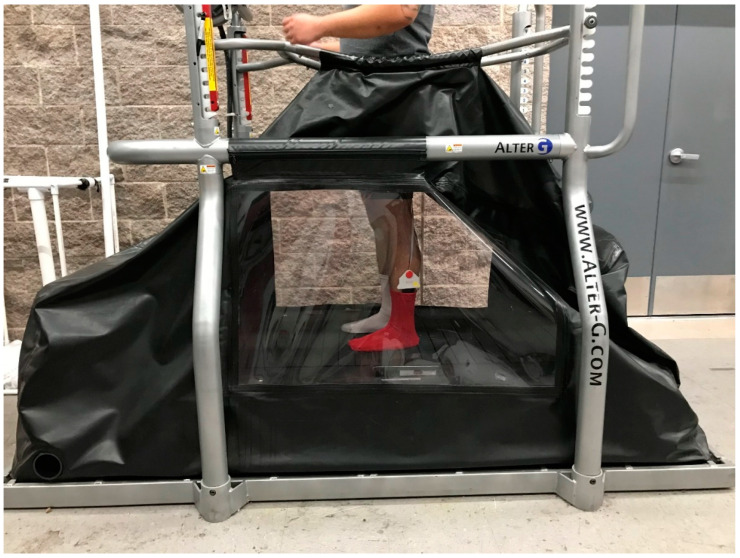
Subject using AlterG^®^ Treadmill with Medilogic^®^ Pressure-Measuring Insoles.

Two instruments were used in this experiment. The simulated reduced-gravity treadmill walking was performed using an AlterG Treadmill (AlterG, Inc., Fremont, CA, USA), Figure 1. The unweighting range of the AlterG is 100% to a minimum of 20% of the user’s body weight. The speed ranges from 0 to 12 mph [25]. 

**Figure 2:**
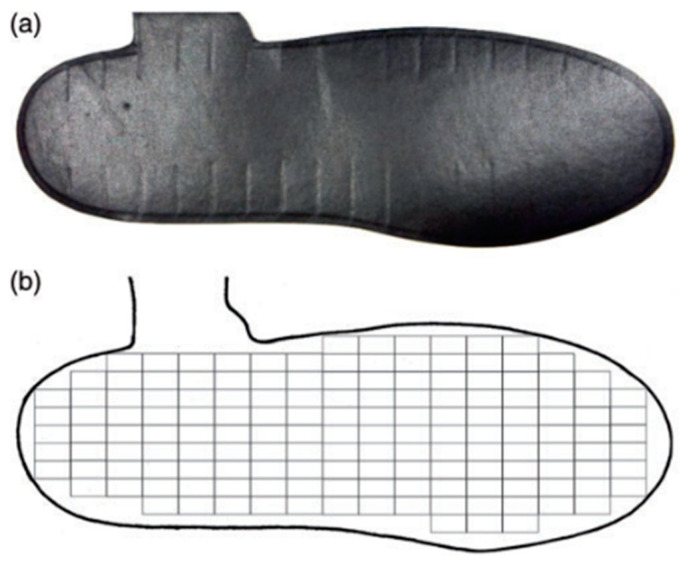
A Medilogic left insole, size 43–44 (**a**) and its corresponding sensor map where each rectangle represents a single sensor (**b**).

The second instrument was the Medilogic (Schönefeld, Germany, 60 Hz) pressure-measuring insole system, Figure 2. These insoles measure plantar pressure data using 1.50 × 0.75 cm rectangular sensors that are arranged to match the plantar area. Six pressure-measuring insole sizes were used in this experiment, with the number of sensors per insole ranging between 93 and 162 (Table 2). A wireless data modem, which was attached to the participant, sends the plantar pressure data from the modem to the computer [26].

### 2.3. Procedures

Each participant was evaluated in a single laboratory visit. Following consent, anthropometric measures were obtained (shoe size, insole size, height, mass, gender, and age). Following the procedures of earlier researchers, participants placed their bare feet on appropriately sized Medilogic pressure-measuring insoles [19,27]. Insoles were taped to the feet to reduce the potential of insole slippage during the experiment. Participants were provided with socks that were worn over the insoles (Figure 1).

To eliminate noise resulting from the pressure generated by the interaction between feet, socks, and insoles, and to set a true zero pressure baseline, the following procedure was implemented at the start of each participant trial:

1. Seated participants were instructed to lift both feet approximately 1–2 inches above the floor.
2. Participants were instructed to stand quietly on the floor with weight equally distributed between the two limbs for a period of 15 s.
3. Participants were instructed again to sit down and lift both feet approximately 1–2 inches above the ground.

The average bit value for both “feet in the air” phases was calculated. This value was labeled as zero bit (ZB).

Participants were then fitted with the AlterG neoprene compression shorts that include a kayak-style skirt that zips into the treadmill aperture to create an airtight seal near the participant’s waist. The AlterG support bars were then aligned to the participant’s iliac crest to ensure appropriate height (Figure 1). The treadmill was then manually calibrated based on the manufacturer’s instructions. Participant-preferred walking speed was determined by setting the treadmill to 100% bodyweight (normal gravity) and the speed to a default of 2.5 miles per hour. Participants were asked to identify a self-selected comfortable walking speed by instructing the researcher on whether to increase or decrease the speed until the value was reached at which they would be walking comfortably for 15 min. This speed was used for three gravity conditions defined as (1) 100% body weight or normal gravity, (2) 40% body weight, and (3) 20% body weight. Each walking condition lasted for 3.5 min. Insole pressure data were recorded during the last 30 s of walking to represent the gait of each participant under the three different gravity conditions. The first 13 steps from each 30-s period were selected to be analyzed.

### 2.4. Plantar Pressure Analysis

Following the procedures of earlier researchers, insole data were processed to obtain the variables of interest for this study [19,27]. The specific dependent variables of interest were COP length (anterior–posterior excursion), COP width (medial–lateral excursion), and stance time. 

Plantar pressure insole output data were recorded as 0–255 bits. The digital output was converted into pressure according to the manufacturer’s suggested conversion scale of 255 bits equal to 64 N/cm² using this formula:

(1) $$p_{ij}=DV_{ij\;}\frac{64\;N/{\mathrm{cm}}^{2}}{255\;\mathrm{bits}}$$

where $DV_{ij}$ is the 0–255 digital output of sensor *i* at instant *j.*

$p_{ij}$ is the pressure of sensor *i* at instant *j.*

The data were evaluated and processed using a MATLAB^®^ (R2019a) custom code. The process started by using a low-pass fourth-order Butterworth filter with a cutoff frequency of 5 Hz to filter the data, which were then zeroed based on the average bit measurement of the noise cancellation phase. The cutoff frequency of 5 Hz was determined through previous experience with the insoles. This value was confirmed through inspection of the results of the data processing code. This filter was able to clearly identify the appropriate number of heel strikes and toe offs based on pressure data for all participants. Some isolated sensors had extremely low bit values due to the pressure of the socks. These sensors were eliminated using an adaptive threshold that identified active sensors in a step by including only sensors whose outputs were above the adaptive threshold of the most significant bit (MSB) instant [28]. This adaptive threshold technique was shown to be more effective in reducing plantar contact area error compared to the fixed-threshold technique. 

The resulting bit data of all sensors were summed at each time instant and used to obtain the normal component of the ground reaction force. Sensors’ readings are summed as

(2) $$F_{Zj}\left(N\right)=\overset{n_{i}}{\underset{i=1}{\sum }}\;p_{ij}\left(1.125\;{\mathrm{cm}}^{2}\right)$$

where $F_{Zj}$ is the vertical component of the ground reaction force at instant *j.*

$n_{i}$ is the number of active sensors on insole *i.*

A 40 N normal force threshold was used to identify heel strike, toe off, and the overall number of steps within each condition [28].

Each sensor’s location and coordinates for each insole size were calculated following the manufacturer’s specifications by determining the *x* and *y* coordinates at the center of each active sensor based on the specific insole sensor map [19]. The active sensors were then used to calculate the coordinates of the COP using the following equation:

(3) $$COP_{x,y_{j}}=\left(\frac{\sum_{i=1}^{n_{i}}p_{ij}\left(x_{i}\right)}{\sum_{i=1}^{n_{i}}p_{ij}},\frac{\sum_{i=1}^{n_{i}}p_{ij}\left(y_{i}\right)}{\sum_{i=1}^{n_{i}}p_{ij}}\right)$$

where *xi* and *yi* are the coordinates of sensor. 

$COP_{x,y_{j}}$ are the coordinates of COP at instant *j*. 

This phase of data reduction produced COP lengths during the first and last segments of the stance phase that were unrealistically long. This may have been a result of the stance thresholds not being based on the COP measurements but rather the ground reaction force values. Therefore, following the procedures of prior researchers, we examined segment lengths during the first or last quarters of the stance. COP length segments that were equal to or greater than 7 mm during the first or last quarter of the stance were identified and eliminated. It was observed that the first and last segment lengths of the stance were unrealistically long. As a result, exceeding tails and hooks that occur at toe off and heel strike may have been due to the resulting signal-to-noise ratio that typically occurs during locomotion. Visual inspections of this tail and hook were also seen from prior researchers in our laboratory. Therefore, we used this criteria threshold as established from prior studies [19]. Stance time measures were obtained as the time between peak force outputs. The average distance between peak force outputs was identified as stance time between each step. In order to compare COP among all participants, COP path length and width was normalized to the respective dimensions, length and width, of each insole. Left and right foot insole data were merged to a combined foot dataset.

### 2.5. Statistical Analysis

Recorded data were analyzed statistically using SPSS software (version 27.0). Two one-way repeated-measures ANOVAs with a Greenhouse–Geisser correction were performed to identify any significant differences between the dependent variables of COP path length and COP path width among each of the three gravitational conditions. Additionally, a one-way repeated-measures ANOVA was also performed to identify any significant differences in stance time measures among each of the three gravitational conditions. The level of statistical significance was set at α = 0.05. 

## 3. Results

Table 3 presents the average and standard deviation values for the COP path length and width, normalized to insole size, for each of the three gravity conditions. ANOVA determined that average COP path length differed significantly among gravity conditions (*p* < 0.05). Post hoc tests using the Bonferroni correction revealed that the 100% BW condition was significantly greater than both the 40% and 20% BW conditions. However, there were no significant differences observed between the 40% BW and the 20% BW conditions (*p* = 0.259). A similar approach was used for COP width. Similar to COP length, ANOVA showed that the average COP path width differed significantly among gravity conditions (*p* < 0.05). Post hoc tests using the Bonferroni correction revealed that the 100% BW condition was significantly less than the 40% and 20% BW conditions. However, there were no significant differences observed between the 40% BW and 20% BW conditions (*p* = 0.134).

Table 3 presents the average and standard deviation values for the stance time measures, normalized to insole size, for each of the three gravity conditions. A repeated-measures ANOVA with a Greenhouse–Geisser correction determined that average stance differed significantly among gravity conditions (*p* < 0.05). Post hoc tests using the Bonferroni correction revealed that the 100% BW condition was significantly greater than both the 40% and 20% BW conditions (*p* < 0.05). In addition, the 40% BW condition was also statistically different from the 20% BW condition (*p* < 0.05). 

## 4. Discussion

In this study, we found that the effect of lower-body positive-pressure treadmill (LBPPT) walking, specifically an increase in body weight support, or a reduction in gravity from 100% (normal gravity) to 40% and 20% (reduced gravity) body weight (BW), is mainly characterized by a significant decrease in center of pressure (COP) anterior–posterior segment length and simultaneous significant increase in COP medial–lateral segment width. The calculated COP location and its movement during the stance phase of each cycle are typically reported as indicators of marked gait stability during dynamic tasks such as walking [20,21]. This alteration in gait stability may be suggestive of a shift in walking patterns as gravity conditions are reduced. The decreasing anterior–posterior length may be suggestive of a shift from a rearfoot strike pattern toward a midfoot or forefoot strike pattern during LBPPT walking. Furthermore, the increasing medial–lateral COP width observed may be indicative of impaired gait stability, as the ability to stabilize the body’s center of mass (COM) over its base of support (BOS) appears to be compromised during LBPPT walking, i.e., an attempt to establish medial–lateral directional stability [29]. 

Center of pressure (COP) measurements have been used by researchers and clinicians to assess static and dynamic postural stability impairments, as in individuals with diabetic peripheral neuropathy or Parkinson’s disease [30,31]. Prior reports have suggested a decrease in COP path length and an increase in COP path width to be predictors of marked instability [18,21,32]. Furthermore, researchers have reported a change in COP during reduced-gravity exercise when compared with normal-gravity exercise [5,33,34,35,36]. 

It is important to note that the decrease in overall dynamic stability as gravity is reduced may be useful for clinicians and researchers, as this change in dynamic stability has been suggested as a possible indicator for fall risk, muscle weakness, and potential gait impairments. In addition, dynamic stability may provide an additional measure to determine when an individual or patient is able to further progress in the rehabilitation process. Therefore, the dynamic stability parameters evaluated in this study may provide important and valuable clinical insight.

Prior reports have demonstrated a reduction in the BOS to significantly increase COP displacement [10]. Although researchers have identified LBPPT walking as a potentially beneficial alternative for rehabilitation purposes due to its ability to reduce musculoskeletal load to recovering tissues, joints, and other biological structures, our findings suggest LBPPT walking may pose a potential compromise to gait stability [8,11]. Perhaps clinicians should advise against LBPPT rehabilitation programs whose primary focus is to rehabilitate postural control and stability impairments. It is suggested that LBPPT may be most clinically useful for those aiming to effectively reduce musculoskeletal load and to facilitate early mobility while simultaneously adhering to safe aerobic exercise. 

The duration of the stance phase during each gait cycle (stance time) was obtained, and a statistically significant difference was found among each of the three gravity conditions. This was particularly interesting, as walking speed was standardized across gravity conditions for each participant, suggesting that stance times should be identical with no concomitant changes in walking kinematics. Because stance time decreased while velocity remained constant, it is feasible to suggest that with reduced gravity, stride rate increased. An increase in stride rate, given a constant velocity, could also suggest a decrease in stride length, as both (stride rate and length) inversely describe walking velocity. These gait characteristics, including step width and cadence, along with gait kinematics, e.g., trunk flexion, hip extension, knee flexion, and ankle dorsiflexion, are commonly evaluated by clinicians and rehabilitation specialists when examining gait abnormalities [13,37]. LBPPT walking may provide a viable instrument for those seeking to adjust or improve specific gait characteristics. The mean difference in stance times during the stance phase could also be indicative of a substantial alteration in gait stability in reduced-gravity environments. We speculate that the effect of LBPPT resulted in a more forefoot or “toe-walking” walking pattern, therefore reinforcing our postulation of a shift in walking strategy. On the contrary, a reduction in stance times would theoretically result in a reduction in compressive forces and ground reaction forces (GRF) exerted by the system, ultimately reducing musculoskeletal load. Hodges-Long et al. [12] found decreased plantar pressure measures during LBPPT running. However, future studies need to examine the degree to which reduced stance times correspond with reductions in GRF during LBPPT walking.

The results of the present study may be useful for clinicians and rehabilitation specialists in the programming of LBPPT exercise. Our results indicate that, at reduced gravity (40% and 20% BW), an increase in instability is observed, as denoted by the increase in COP path width. Therefore, our findings suggest a potential threshold to which clinicians and specialists should perhaps prescribe LBPPT BW levels to the recovering individuals and patients. Future work is necessary to further confirm our findings and provide additional findings to further elucidate this potential threshold of gravity conditions.

Various limitations should be considered when interpreting the results of this study. Although plantar pressure data were recorded during the last 30 s of each walking condition, participants were not provided an accommodation time or learning phase for each of the gravitational walking conditions beyond the initial 2.5 min of walking prior to data collection. Variability in plantar pressure measures may have resulted from a lack of familiarity with LBPPT walking. Variability may have also occurred due to the relatively small sample size, perhaps resulting in type II error. In addition, the order in which participants performed the three walking trials, i.e., starting at 100% (normal gravity) BW, then 40%, and finally 20%, may have resulted in participant muscle fatigue. This fatigue may have contributed to the increased instability, as indicated by the decrease in COP path width. Lastly, we did not examine other gait parameters, such as stride length, step length, step width, etc., given the physical space limitations of the treadmill housing the pressurized air chamber combined with the specific focus of the present study, which was dynamic stability during reduced-gravity locomotion. 

## 5. Conclusions

The purpose of the study was to investigate the effect of LBPPT walking on selected gait parameters in simulated reduced-gravity conditions. Our results indicated that as gravity was reduced, COP length increased and COP width decreased, while stance time decreased with a reduction in gravity. As well, the pattern of gait migrated for many participants for what is observed normally to be a toe-walking form of ambulation.

Future research should consider the use of a familiarization or accommodation period with respect to LBPPT walking. In addition, a larger sample size to strengthen the effect of plantar pressure measures performed in reduced-gravity locomotion may provide greater insight into LBPPT walking. Additionally, thresholds of reduced gravity more closely mimicking full body weight (e.g., 80%, 60%) should be investigated to identify a more clinically feasible level of body weight reduction to allow for appropriate lower extremity loading while avoiding a migration to a toe-walking pattern of gait.

## Figures and Tables

**Table 1:** Demographic variables of participants.

European Insole Size	Number of Participants	Age (Year)	Mass (kg)	Height (m)	Gender
35–36	5	22.8 ± 3.96	53.02 ± 6.15	1.53 ± 0.04	Male (0) Female (5)
37–38	7	23.1 ± 3.85	56.49 ± 8.39	1.60 ± 0.05	Male (0) Female (7)
39–40	5	26.2 ± 2.59	77.04 ± 18.77	1.65 ± 0.04	Male (1) Female (4)
41–42	3	26.7 ± 3.79	79.5 ± 18.19	1.74 ± 0.01	Male (1) Female (2)
43–44	4	24.5 ± 5.00	76.45 ± 15.49	1.69 ± 0.06	Male (4) Female (0)
45–46	5	26 ± 5.20	91.68 ± 14.15	1.85 ± 0.06	Male (5) Female (0)

**Table 2:** Number of sensors per insole.

Insole Size	Number of Sensors
35–36	93
37–38	107
39–40	116
41–42	130
43–44	151
45–46	162

**Table 3:** Normalized average and standard deviation COP path length, COP path width, and stance time across each gravity condition.

Gravity Condition	COP Path Length	COP Path Width	Stance Time
100% BW	0.626 ± 0.076	0.109 ± 0.023	0.777 ± 0.071
40% BW	0.438 ± 0.223 ^1^	0.152 ± 0.037 ^1^	0.753 ± 0.096 ^1^
20% BW	0.383 ± 0.190 ^1^	0.162 ± 0.043 ^1^	0.703 ± 0.126 ^1,2^

^1^ Significantly different (*p* < 0.05) from 100% BW. ^2^ Significantly different (*p* < 0.05) from 40% BW.
